# Bias in Diet Determination: Incorporating Traditional Methods in Bayesian Mixing Models

**DOI:** 10.1371/journal.pone.0080019

**Published:** 2013-11-05

**Authors:** Valentina Franco-Trecu, Massimiliano Drago, Federico G. Riet-Sapriza, Andrew Parnell, Rosina Frau, Pablo Inchausti

**Affiliations:** 1 Proyecto Pinnípedos, Sección Etología, Facultad de Ciencias, Universidad de la República, Montevideo, Uruguay; 2 Department of Animal Biology, Faculty of Biology, University of Barcelona, Barcelona, Spain; 3 School of Mathematical Sciences (Statistics), Complex Adaptive Systems Laboratory, University College Dublin, Dublin, Ireland; 4 Departamento de Ecología y Evolución, Centro Universitario Regional Este (CURE), Universidad de la República, Maldonado, Uruguay; University of California, Berkeley, United States of America

## Abstract

There are not “universal methods” to determine diet composition of predators. Most traditional methods are biased because of their reliance on differential digestibility and the recovery of hard items. By relying on assimilated food, stable isotope and Bayesian mixing models (SIMMs) resolve many biases of traditional methods. SIMMs can incorporate prior information (i.e. proportional diet composition) that may improve the precision in the estimated dietary composition. However few studies have assessed the performance of traditional methods and SIMMs with and without informative priors to study the predators’ diets. Here we compare the diet compositions of the South American fur seal and sea lions obtained by scats analysis and by SIMMs-UP (uninformative priors) and assess whether informative priors (SIMMs-IP) from the scat analysis improved the estimated diet composition compared to SIMMs-UP. According to the SIMM-UP, while pelagic species dominated the fur seal’s diet the sea lion’s did not have a clear dominance of any prey. In contrast, SIMM-IP’s diets compositions were dominated by the same preys as in scat analyses. When prior information influenced SIMMs’ estimates, incorporating informative priors improved the precision in the estimated diet composition at the risk of inducing biases in the estimates. If preys isotopic data allow discriminating preys’ contributions to diets, informative priors should lead to more precise but unbiased estimated diet composition. Just as estimates of diet composition obtained from traditional methods are critically interpreted because of their biases, care must be exercised when interpreting diet composition obtained by SIMMs-IP. The best approach to obtain a near-complete view of predators’ diet composition should involve the simultaneous consideration of different sources of partial evidence (traditional methods, SIMM-UP and SIMM-IP) in the light of natural history of the predator species so as to reliably ascertain and weight the information yielded by each method.

## Introduction

Trophic interactions determine the flow of energy among trophic levels that ultimately drives population dynamics, community and food web structure and most ecosystem processes. Determining the diet composition of predators in a community is essential to measure the potential niche overlap and the strength of their interspecific interactions [1]. There are no “universal methods” allowing the reliable determination of the diet composition of predators. In the case of vertebrate, diet composition can rarely be determined through the direct observation of prey consumption [2,3]. Ecologists must then employ several indirect methods to determine diet composition. These methods include the analyses of stomach contents [3,4], scats [5–7], regurgitations [8] and pellets [9,10]. Nevertheless, all these indirect methods rely on the recovery and identification of hard items (e.g. otholites, beaks, bones) of consumed preys, but yield high-resolution information on the species composition, body length and mass of the prey consumed [10]. However, all these traditional methods possess intrinsic biases mostly due to differential digestion, retention and recovery of preys’ hard items that can severely biased estimates of diet composition when the latter contains preys that are either fully digested or lack such hard items [6,11,12]. Scat analysis is perhaps the least invasive of the traditional, indirect methods to determine diet composition and probably the one yielding the highest sample size for the lowest effort and cost [13]. 

Others indirect methods for diet determination in vertebrates do no depend on the recovery of hard items of the preys consumed. These alternative indirect methods include molecular identification [14], signature fatty acids analysis [15] and stable isotope analyses [16]. In the last decades, stable isotope (^13^C and ^15^N) analysis has become a standard tool in the study of the foraging ecology of vertebrate [17–20]. Stable isotope analysis can provide indirect but otherwise unobtainable information on habitat and resource use [21]. The use of stable isotopes to determine diet composition is based on three assumptions: (1) carbon and nitrogen contents come directly from the ingested food and hence they can be used to ascertain the relative importance of potential preys [22,23]; (2) the isotopic signal of each tissue depends on its metabolic rate and thus the time reference of dietary information is tissue-dependent [24]; and (3) the assimilated organic compounds are enriched in the heavy isotopes (^13^C, ^15^N) after transference between consecutive trophic levels [25]. This differential enrichment occurring in the conversion from proteins of preys to the consumer tissues is called fractionation or trophic discrimination factor and, by varying among taxa and tissues [26], it can substantially affect the estimates of diet composition. 

The recent development of mixing models has allowed their use to obtain diet composition based on isotopic contents of predators’ tissues and their potential preys [27,28]. Mixing models aim to obtain the relative contribution of all preys consumed to a predator’s diet [28]. Recent developments of mixing models (MixSIR [29]:; SIAR [27]:) based on Bayesian methods allow considering many potential preys and can deal with the uncertainty inherent in isotopic measurements. Bayesian stable isotope mixing models (SIMM) can incorporate prior information (i.e. either the proportional prey consumption or the diet composition as determined by traditional methods) that often lead higher precision in the estimated dietary composition than when the latter is obtained using uninformative priors [27,29]. Therefore, both the diet composition and the precision of the estimated contribution of each prey species can be affected by the uncertainty of inputs to SIMMs [29]. To our knowledge, there have been few comparisons of the estimated diet compositions obtained using SIMMs with more traditional methods of diet determination [30–32]. We believe that both traditional methods such as stable isotope analysis have different biases associated with its interpretation to determining the diet composition of a species [33]. For this reason, the combination of both techniques through SIMMs seems a promising approach to estimate the diet composition because their use of prior information allows incorporating different sources of variability as input in the analysis [33]. The goal of this paper is to evaluate the biases and precision of three commonly used methods (scat analysis, SIMM with and without informative priors) to study predators’ diet composition using the South American fur seal (SAFS, *Arctocephalus australis*) and sea lions (SASL, *Otaria flavescens*) as a study system 

## Materials and Methods

### Study area and sample collection

The study was conducted at Isla de Lobos, one of the main breeding colony of SASL and SAFS in Uruguay (35° 01' S; 54° 52' W), located at five nautical miles from the mainland. Females of SAFS and SASL have colonial breeding while males fast while defending territories for mating. Therefore, scats collected in the rookery at this time can only reflect the feeding by breeding females. Two hundred twenty-seven SAFS scats and 52 SASL scats [34] were collected during the breeding season in December/2006 and January/2009, respectively. Scats samples were washed through a 0.5 mm mesh sieve at the laboratory; prey hard items recovered were compared with reference collection [35,36] and an otoliths reference collection (Franciscana Project, Facultad de Ciencias, University of República, Uruguay). Otoliths used to estimate diet composition were only those having minimal erosion that allowed prey identification to species level and cephalopod beaks were identified to the lowest possible taxon; hard items digested beyond recognition were not included in the analysis. 

During the same breeding seasons when scats samples were collected, we gather skin samples from the caudal flippers from randomly selected lactating SAFS (n=35) and lactating SASL (n=10). Skin samples were stored in the field and were used for isotope analysis in the lab. We obtained skin samples from the caudal flippers for isotope analysis from randomly 35 lactating SAFS and 10 lactating SASL selected during same breeding seasons. Lactating females were captured with a hoop net and sedated using ~2ml of Midazolan 0.5% in the case of SAFS, while SASL were anaesthetized using isoﬂurane gas mixed with oxygen (0.5–2.5%) using a portable-ﬁeld vaporizer [34]. The present research was conducted under the permits 603/2006 and 572/2008 approved by DINARA (National Administration of Aquatic Resources), Ministry of Livestock, Agriculture and Fisheries, Uruguay. All procedures of animal manipulation were submitted and approved as valid according to the national laws in animal welfare by the Ethics Committee in Animal Experimentation, Universidad de la República, Uruguay. Skin samples were dried in a stove at 60°C for 36 h, and lipids extraction was made as Bligh and Dyer [37]. Approximately 0.3 mg of skin without lipids were weighed into tin cups (3.3 x 5 mm), combusted at 900°C, and analysed in a continuous flow isotope ratio mass spectrometer and stable isotope abundances are expressed in delta (δ) notation. Samples were processed at Stable Isotope Laboratory of the University of República (Uruguay) with an analytical error estimated in 0.1‰ for nitrogen and 0.03‰ for carbon. Isotopic values of potential prey species in waters of the Río de la Plata and the Uruguayan continental shelf were obtained from Franco-Trecu et al. [20] and were used in the Bayesian mixing models based on stable isotopes.

### Diet composition from scat analysis and using SIMMs on stable isotopes

We characterized the diet composition of each predator species by the relative abundance of each species of fish or cephalopod in the set of scat samples for each predator species. The number of individuals of each fish and cephalopods species found in each scat sample was calculated following [38]. Diet composition in the scat analysis was estimated by its relative numerical abundance of each prey species across all scats samples of each predator species. To have analogous estimates of the estimated diet composition and its variability for all method used, we calculated the average and the percentiles of the importance of each prey species in the diets by bootstrapping the matrices of results for each predator species using a sampling size equal to the number of scats observed with at least one hard and using 10,000 iterations [39].

We also estimated diet composition of each predator species using the SIMMs in the SIAR (Stable Isotope Analysis in R) library [27] of the R free software [40] with and without informative prior distributions based on the relative abundance of each prey species estimated from the scat analysis. The input of the SIMMs [27] comprised the δ^13^C and δ^15^N values from the all skin samples of SAFS and SASL females, the mean and standard deviation for their potential prey species (selected according to the results of the scat analysis) in the Uruguayan marine ecosystems, and the mean and standard deviation of prey elemental concentration (C and N) and we used skin trophic enrichment factor obtained from [41]. 

For both SIMMs with and without informative priors, we used the Dirichlet multinomial distribution to define these prior distributions. In the case of the SIMM with uninformative prior (SIMM-UP), the mean and variance defining the Dirichlet distribution were 1/k and (k-1)/k^2^*(k+1) with k being the number of potential preys of SASL and SAFS [27]. In the SIMM with uninformative prior (SIMM-IP), the mean and variance of the prior Dirichlet distribution were obtained from the relative numerical abundances of potential preys of SAFS and SASL and the standard deviation of the relative abundance of one of these potential prey species [27]. We chose the Striped weakfish (*Cynoscion guatucupa*) to SAFS and Lergehead hairtail (*Trichiurus lecturus*) to SASL. The SIMM output gives the diet composition as the posterior probability distribution having absorbed the prior information (or lack thereof) and the likelihood function containing a probability model and the isotopic data [27]. Each SIMM was based on 500,000 iterations, thinned by 15 and with an initial discard of 50,000 iterations, resulting in 30,000 posterior draws of the posterior distribution. Convergence to the posterior distribution in each model was assessed by the Geweke’s criterion [42] and was shown to be acceptable for all models here considered. We compared the similarity in the diet compositions obtained by the scat analysis and the SIAR with and without informative priors using Bhattacharyya’s Coefﬁcient [43]. This coefficient of similarity between sampling distributions is analogous to other indices of diet or niche overlap (e.g. Horn-Morisita), taking values between 0 (completely different diets) and 1 (equal diets) [44].

## Results

**Figure 1:**
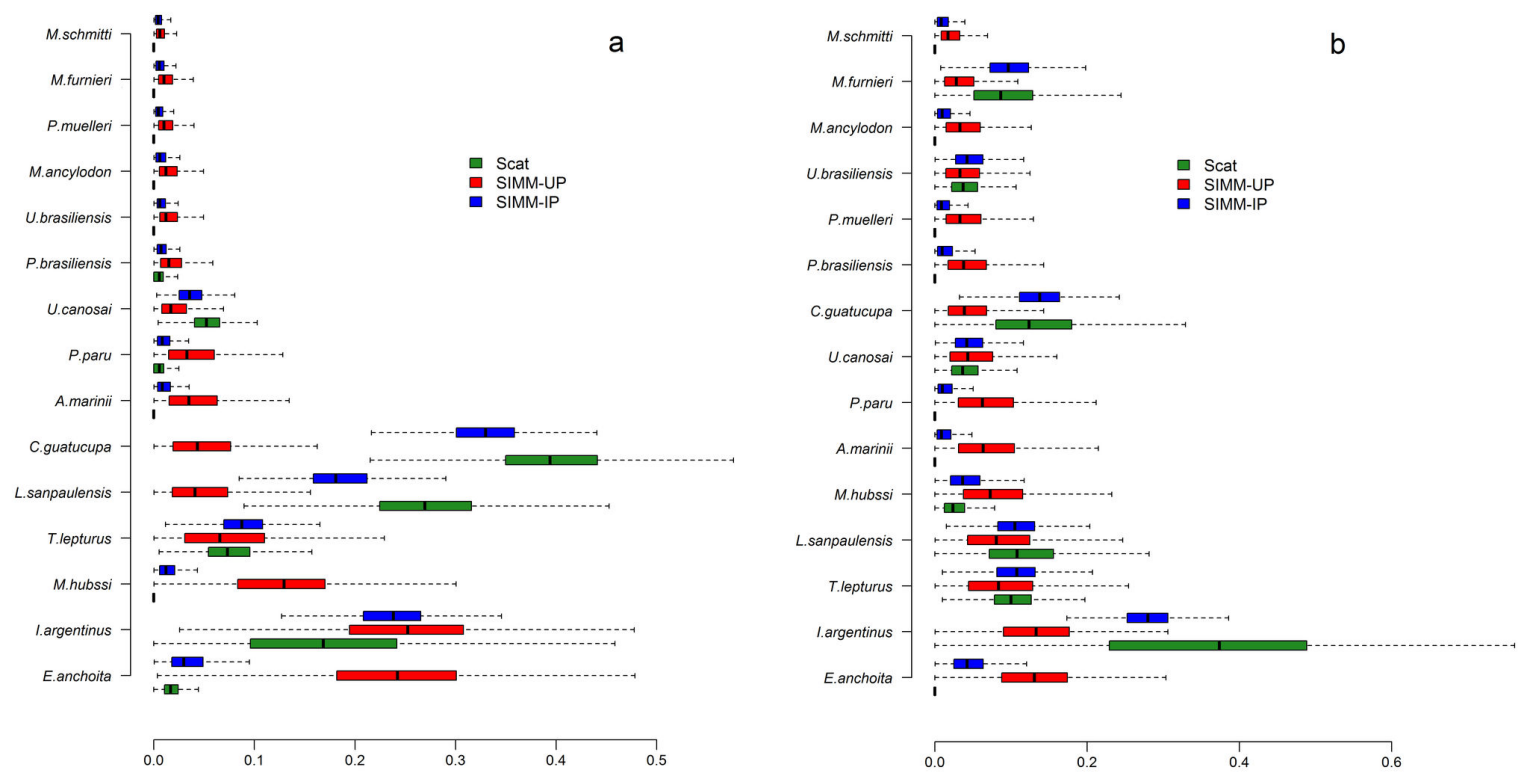
Diet composition comparison by scat and Bayesian mixing models with and without prior information. Diet composition of the South American fur seal (*Arctocephalus australis*) (a) and South American sea lion (*Otaria flavescens*) (b) in Isla de Lobos, Uruguay estimated by scat analysis (light grey bars), Bayesian mixing modes with uninformative (SIMM-UP; dark grey bars) and informative (SIMM-IP; black bars) priors. Mixing models were obtained with the library SIAR in the R software [27]. The error bars for the scat analysis were obtained by bootstrap.

We found prey hard parts only in 37% of the SAFS (94 otoliths and 77 cephalopods beaks) and in 73% of the SASL scats samples (39 otoliths and 40 cephalopods beaks).The most abundant identified preys in the scats of SAFS was Striped weakfish (38%) and in SASL was the Argentine shortfin squid (36%) (Table 1, Figure 1a, b). According to the SIMM-UP, more than 60% of SAFS’s diet was composed by pelagic species such as Argentine hake (*Merluccius hubbsi*), Argentine anchovy (*Engraulis anchoita*) and Argentine shortfin squid (Figure 1a), whereas that of SASL was more diverse and even without any clear dominance of the consumed preys (Table 1, Figure 1b). In contrast, the bulk of the diets of both predators estimated by SIMM-IP tended to be dominated by the same preys showed to be important in the scat analysis (Figure 1a, b, Table 1). Although including informative priors in the SIMM improved the precision (i.e. decreased the width of the 95% CI for each prey) of the estimated diet compositions of each predator compared with those obtained with uninformative priors (Figure 1a, b), the results maybe do not look more reasonable.

**Table 1:** Scat and Bayesian mixing models diet composition.

**Prey species**	**SAFS**			**SASL**		
**Common and scientific name**	**Scats**	**SIMM-UP**	**SIMM-IP**	**Scats**	**SIMM-UP**	**SIMM-IP**
**Brazilian codling (*Urophysis brasiliensis*)**	-	0.02 (0-0.05)	0.01 (0-0.02)	0.04	0.04 (0.01-0.09)	0.05 (0-0.1)
**Whitemouth croaker (*Micropogonias furnieri*)**	-	0.01 (0-0.04)	0.01 (0-0.02)	0.09	0.04 (0.05-0.15)	0.1 (0.03-0.16)
**Striped weakfish (*Cynoscion guatucupa*)**	0.38	0.05 (0-0.14)	0.33 (0.24-0.4)	0.13	0.05 (0.08-0.21)	0.14 (0.07-0.22)
**Argentine croaker (*Umbrina canosai*)**	0.05	0.02 (0-0.07)	0.04 (0.01-0.07)	0.04	0.05 (0.01-0.09)	0.05 (0-0.1)
**Banded croaker (*Paralonchurus brasiliensis*)**	0.01	0.02 (0-0.06)	0.01 (0-0.02)	-	0.05 (0-0.04)	0.01 (0-0.05)
**American harvestfish (*Peprilus paru*)**	0.01	0.04 (0-0.11)	0.01 (0-0.03)	-	0.07 (0-0.04)	0.01 (0-0.05)
**King weakfish (*Macrodon ancylodon*)**	-	0.02 (0-0.05)	0.01 (0-0.02)	0.01	0.04 (0-0.04)	0.01 (0-0.04)
**Red Shrimp (*Pleoticus muelleri*)**	-	0.01 (0-0.04)	0.01 (0-0.02)	-	0.04 (0-0.03)	0.01 (0-0.04)
**Narrownose smooth-hound (*Mustelus schmitti*)**	-	0.01 (0-0.02)	0 (0-0.01)	-	0.02 (0-0.03)	0.01 (0-0.03)
**Argentine hake (*Merluccius hubbsi*)**	-	0.13 (0.01-0.23)	0.01 (0-0.03)	0.03	0.08 (0-0.08)	0.04 (0-0.09)
**Marini's anchovy (*Anchoa marinii*)**	-	0.05 (0-0.12)	0.01 (0-0.03)	-	0.07 (0-0.04)	0.01 (0-0.05)
**Argentine anchovy (*Engraulis anchoita*)**	0.02	0.25 (0.08-0.42)	0.04 (0-0.07)	0.04	0.13 (0.01-0.08)	0.05 (0-0.1)
**Largehead hairtail (*Trichiurus lepturus*)**	0.08	0.08 (0-0.18)	0.09 (0.04-0.14)	0.10	0.09 (0.05-0.17)	0.11 (0.04-0.18)
**Argentine shortfin squid (*Illex argentinus*)**	0.18	0.25 (0.08-0.41)	0.25 (0.18-0.32)	0.36	0.13 (0.23-0.35)	0.28 (0.2-0.35)
**Sao Paulo squid (*Loligo sanpaulensis*)**	0.27	0.05 (0-0.14)	0.18 (0.11-0.12)	0.11	0.09 (0.05-0.17)	0.11 (0.04-0.18)

Diet composition of the South American fur seal (SAFS, *Arctocephalus australis*) and South American sea lions (SASL, *Otaria flavescens*) in the summer of 2006 and 2009 respectively estimated by the scat analysis (expressed as the proportion of the prey individuals across all individuals in total scat samples) and by the Bayesian mixing models with (SIMM-IP) and without (SIMM-UP) informative priors (showing the mean and 95% CI for each prey).

There were important differences in the estimated diet composition of SAFS when informative priors were included. Estimates of diet composition obtained scat analysis and SIMM-UP had a much smaller similarity (BC coefficient) for SAFS (68%) than for SASL (89%). The similarity of the diet compositions estimated by scat analysis and SIMM-IP was greater than 96% for both species, whereas those of the SIMMs had a similarity of 83% and 92% for SAFS and SASL, respectively. The differences in estimated SAFS diets between scat and SIMM-UP were mostly due to the marked decrease in relative importance of the Argentine anchovy and Argentine hake decreased, while the importance of Stripped weakfish and Sao Paulo squid (*Loligo sanpaulensis*) increased (Figure 1a). A similar comparison for SASL showed both the increase in the relative importance of Stripped weakfish, Whitemouth croaker (*Micropogonias furnieri*) and Argentine shortfin squid and the decline in the importance of Argentine and Marini’s anchovy (*Anchoa marinii*) (Figure 1b).

## Discussion

The development of SIMMs to determine the diet composition and the strengths of trophic interactions based on stable isotopes has led to stream of publications over the past years [33]. Recently, research on the SIMMs have addressed the effect of uncertainties in the discrimination factor of each tissue and species [32,45], the number of stable isotopes used [46] and on whether lipids need be extracted in samples [47]. Being based on Bayesian methods, SIMMs must incorporate priori knowledge on diet composition as informative priors that can be obtained by different means, including assessing the relative prey consumption from their relative abundance in the environment [48], expert opinion and on several indirect methods of estimating diet composition [27]. However, to our knowledge, there has been little research (see 29) comparing the diet compositions simultaneously estimated by traditional indirect methods and by SIMMs with and without informative priors. 

**Figure 2:**
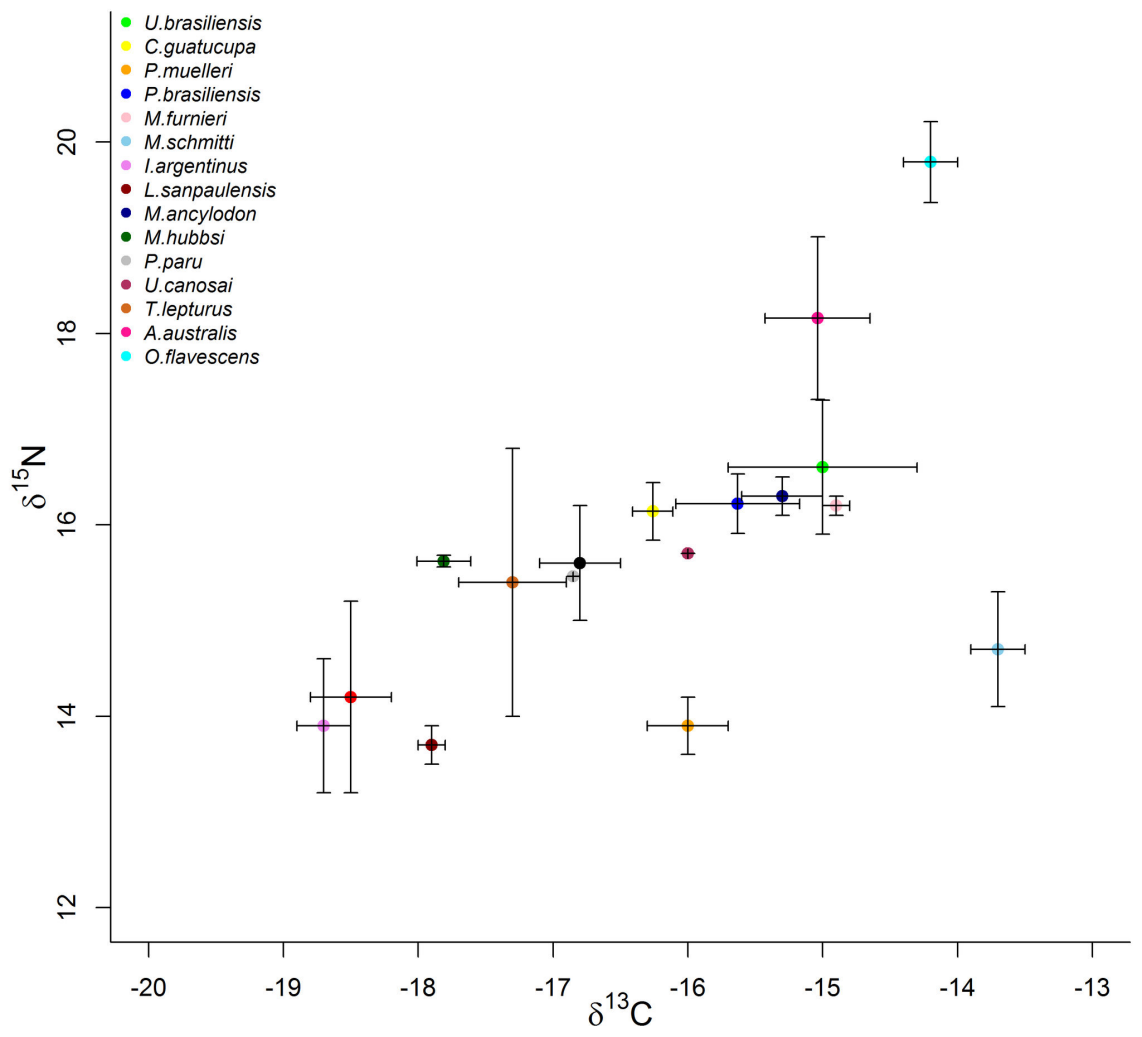
Predator and potential preys’ stable isotope signal. Biplot of the isotopic contents of δ^15^N and δ^13^C of the South American sea lion (*Otaria flavescens*), the South American fur seal (*Arctocephalus australis*) and their main potential preys in Uruguay. Prey species were captured in the pelagic and neritic areas of the Uruguayan continental shelf and their names are fully indicated in Table 1. Error bars correspond to standard deviations. These averages and standard deviations were used as input for the mixing models.

One of well-known biases of traditional indirect methods of diet estimation comes from the non-detection of preys lacking hard remains. In our case, however, SIMMs did not show that soft-bodied species (e.g. Red shrimps, Narrownose smooth hounds) were at all important in the diet compositions of either predator species. Thus, while scat analysis and SIMMs agreed on which preys composed each predator’s diet, they differed in the relative importance of each prey species, with the difference being very pronounced for the SAFS but not for SASL (see Figure 1). When compared with other mixing models, SIMMs do incorporate different sources of uncertainty occurring in the data, they should not be expected to estimate diet composition with reasonable precision when there is only a moderate information content in the data [29,48]. The increase in precision in diet composition may sometimes come at the price of obtaining biased estimation of diet composition. Including biased prior information into SIMMs may lead to biased posterior distribution depending on the nature of input data [33]. It is well known in Bayesian analysis that prior information may strongly affect the posterior distribution only when the data contain a modest amount of information allowing discriminate single prey contributions to the diet [29]. The latter would happen when prey isotopic contents have a large overlap in isotopic space [48–50], as it is the case of Argentine anchovy and Argentine shortfin squid (Figure 2). In such cases where overlapping isotopic data does not allow differentiating the relative contribution of each prey species, the SIMM-UP renders each of these preys have a similar importance in the predators’ diets, with a relative large uncertainty shows as a wide CIs (Figure 1). The reduction of the widths of 95% IC after including informative prior (Figure 1) was probably due to the availability of other sources of information that allowed discriminating between preys having similar isotopic values. Therefore, one must pay particular attention to the potential biases associated with the prior information in SIMMs because the latter are bound to both reduce and increase the importance of certain prey species in a predator’s diet (see Figure 1). 

Ideally, prior information would convey the potential (or known) prey consumption, which involves knowing the actual prey abundances as perceived by predators and their selectivity or diet preferences. On a technical level, the elicitation of prior information for compositional data like that input to SIMM is an ongoing research problem. The Dirichlet prior used in SIMM requires the input of mean proportion estimates for each food source, and a standard deviation estimate for a single one of these. Clearly this is not necessarily using all of the information that may be available (e.g. standard deviations for other food sources). One option is to use the product of individual Beta distributions [29], though this has the unfortunate side effect that prior specifications for different food sources may conflict with each other. The most promising way forward appears to be the use of log-ratio transforms, for which all quantities can be specified without any conflict (Parnell et al. 2013), though this is yet to be incorporated in any of the widely software packages. Obtaining prior information based on empirical data with reasonable accuracy and precision is rather difficult (if not impossible) for most predator species and habitats. Scat analysis and other traditional methods estimating diet composition seem to be the only practical means by which ecologists may obtain data that can be used as informative priors in SIMMs [29]. The issue is then how to discern whether these informative priors actually lead to biased posterior estimates of diet composition. 

We believe that the only answer to the potential biases induced by prior information in SIMMs can come from using extensive knowledge on the natural histories of studied species and of habitats so as to interpret potential differences in the posterior distributions arising from using different priors [50]. Our studied objects were two sympatric pinnipeds species breeding in rookeries along the Uruguayan coast. Summer breeding involves a long lactation period lasting approximately 11 months for both species during which mothers alternate their foraging trips at sea with suckling bouts on land [51]. Both species mostly forage in the Uruguayan continental shelf (an extended and shallow area of approximately 200 km wide) and the shelf break. The foraging trips of lactating SASL females last only a few days and they largely remain in nearshore areas [34]. In contrast, SAFS females mainly forage in offshore areas [20] carrying out long foraging trips often lasting around 15 days [52] and traveling up to 500km away from the rookery (Franco-Trecu unpublished data). These differences in foraging behaviour between SAFS and SASL could lead to other biases in the estimated diet composition when scat analysis is used as prior information in SIMMs. 

Given that the gut transit time in pinnipeds is at most five days [53] and that SAFS females forage for many days and far away from the rookery, many scats collected upon their return were empty [5] as in this study. Therefore, preys consumed by SAFS farther away from the rookery may be represented with low frequency or be altogether absent in scats, which would result in a biased estimation of SAFS diet composition towards those prey species consumed near the rookery. The latter may explain why the similarity of SAFS diet composition obtained with SIMM-UP and scats was much smaller that for all other paired comparisons. The SIMM-UP showed that SAFS’s main preys were pelagic species (see Figures 1, 2) that can mostly be found far away from the rookery [54,55], thus matching both the foraging areas used by SAFS and the long duration of its foraging trips [52]. However, incorporating informative priors in SIMM shifted the rankings of preys such that Stripped weakfish represents about one-third of SAFS diet, an importance very similar to that obtain from the scat analysis. The Stripped weakfish is a very abundant species [54,55] that is one of the main targets of commercial fisheries in the Uruguayan continental shelf. On these grounds, Stripped weakfish ought to be well represented in SAFS scats and its high importance in the diet estimated by SIMM-IP suggests that SAFS mostly consumes this prey while returning to the rookery. In contrast, by foraging in areas close to the rookery and having short foraging trips, SASL scats are likely to contain representative samples of the preys consumed by this species. 

 Traditional indirect methods of diet composition (i.e. scat, pellet and gut contents) typically require intensive effort over time to obtain representative samples of the preys consumed. Also, differential digestibility of preys and their reliance on recovering hard items make these methods prone to both under- and over-estimate the importance particular preys in the diet [6,12]. In contrast, stable isotope analysis require more easily obtained samples depending on the tissue analysed and the SIMMs can resolve many problems related to bias due to the different digestibility of preys [56] because they only consider assimilated food. However, SIMMs have other problems such as their sensitivity to have correct and accurate fractionation factors for the tissue and species analysed. Nevertheless, compared with other indirect methods, the information generated by stable isotopes seems at present the most reliable method to determine diet composition. While some studies have shown that diet composition obtained from traditional indirect methods coincide with SIMMs [30,57], this agreement is far from universal [31,58], leading to disparities in estimated length of trophic chains, trophic level and diet diversity [59]. For instance, the importance of trash in seagulls’ diet and of fish discards in other seabirds is often found by traditional methods [30] but SIMMs can hardly ascertain its importance because of the near impossibility of assigning a unique isotopic content [46]. 

Both SIMMs and traditional indirect methods seem to have complementary strengths and limitations that almost always yield a partial understanding of diet composition. In cases when prior information influences the posterior distribution of SIMMs, incorporating informative priors should almost always lead to improvements in the estimates of diet composition at the risk of inducing biases in the contributions of prey species to the diet. Therefore, just as estimates of diet composition obtained using traditional indirect methods need be critically interpreted because of their known biases, care must be exercised when interpreting diet composition obtained by SIMMs with informative priors. However, as preys isotopic data allow a better discriminatory power of preys’ contributions to the diet, using informative priors should lead to more precise but largely unbiased estimates of diet composition. This should be particularly important for species living in very productive habitats with a high diversity of potential preys having similar signals in the isotopic space [46]. We believe that the best approach to obtain a near-complete view of predators’ diet composition should involve the simultaneous consideration of different sources of partial evidence (traditional methods and SIMM with and without informative priors) in the light of known natural history of the predator species under study so as to reliably ascertain and weight the information yielded by each method. 
